# Assessment of Work‐Related Musculoskeletal Disorders and Ergonomic Risk Factors Among RMG Workers Using REBA and NMQ: A Public Health Study in Savar and Gazipur, Bangladesh

**DOI:** 10.1002/puh2.70237

**Published:** 2026-04-13

**Authors:** Md. Mehedi Hasan, Jannatul Hayat Rima, Hafsa Hasan Jannat, Md. Yeasir Arafath Jisan, Md. Nazmul Haque Nayem, Md. Mejbah Uddin Mithu

**Affiliations:** ^1^ Department of Public Health Daffodil International University Savar, Dhaka Bangladesh

**Keywords:** ergonomic assessment, musculoskeletal disorders (MSDs), Nordic Musculoskeletal Questionnaire (NMQ), occupational health, public health, Rapid Entire Body Assessment (REBA), ready‐made garment (RMG) workers

## Abstract

**Background:**

Work‐related musculoskeletal disorders (WMSDs) represent a notable public health issue among ready‐made garment (RMG) workers, especially in developing countries where ergonomic hazards are frequently neglected. This study sought to evaluate the prevalence, risk factors, and ergonomic hazards related to WMSDs among RMG laborers in Bangladesh.

**Methods:**

A cross‐sectional study was carried out involving 656 RMG laborers from Savar and Gazipur, Bangladesh. Data were gathered through the use of the Nordic Musculoskeletal Questionnaire (NMQ) and the Rapid Entire Body Assessment (REBA) instrument. Demographic, occupational, and ergonomic variables were examined through chi‐square tests and logistic regression analysis.

**Results:**

The high prevalence over the past 12 months was observed in lower back pain at 39.5%, followed by neck pain at 33.5% and ankle pain at 33.5%. Female employees, those engaged in needlework activities, and individuals working overtime or maintaining extended periods of seated posture exhibited significantly higher likelihood of developing WMSDs. Among the REBA subsample (*n* = 43), more than 53% were classified as high or very high ergonomic risk. Significant correlations were identified between WMSDs and variables including gender, work section, posture, overtime, and work experience (*p* < 0.05).

**Conclusion:**

WMSDs are extensively prevalent among Bangladeshi RMG laborers, with ergonomic and occupational factors serving as key contributors. Immediate ergonomic interventions, policy modifications, and worker education are essential to mitigate WMSD risks and enhance worker health and productivity.

## Graphical abstract

A cross‐sectional study among 656 Bangladeshi RMG workers found high 12‐month prevalence of lower back (39.5%), neck (33.5%), and ankle (33.5%) disorders. Female gender, prolonged sitting, overtime, and ergonomic risk (REBA) were significantly associated, highlighting urgent need for workplace ergonomic interventions.

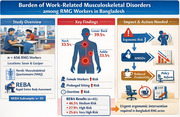

## 1 Introduction

Work‐related musculoskeletal diseases (WMSDs) are becoming more widespread, and occupational health risks are not unusual in workplaces [1]. Workers are more susceptible to musculoskeletal disorders (MSDs) when they are obliged to operate at workstations that are not ergonomically suitable [2]. Nowadays, visiting attendance to a doctor is among them MSDs, the second leading cause [3]. The term “musculoskeletal disorders” (MSDs) refers to injuries or conditions that impact the musculoskeletal system, which includes the bones, muscles, tendons, ligaments, nerves, discs, blood vessels, and tissues. Among the MSDs are carpal tunnel syndrome, epicondylitis, thoracic outlet syndrome, tendonitis, and muscle/tendon strain, lower back pain, neck pain, frozen shoulder, trigger finger, or stenosing tenosynovitis [2]. There are several departments in a ready‐made garment (RMG) factory. Workers in the sewing, cutting, finishing, ironing, and packing industries are frequently at risk for work‐related musculoskeletal disorders (WMSDs), which can be brought on by the manual and repetitive nature of their jobs. The risk factor for growing MSDs is poorly designed workstation such as nonadjustable seat heights and backrests, quality of machine, height of table, vibrations and noise made from machine, and adaptation of prolonged awkward posture also; prolonged working hours, nonergonomic weight‐lifting motions, and repetitive motions causing physical and mental fatigue. Moreover, personal factors such as fitness, age, and previous health also play a role in developing MSD [1, 4, 5]. The International Labor Organization reports that there are 160 million work‐related illnesses that occur each year. [6] In LMICs (low–middle‐income countries), MSDs are not given as much priority. In Ghana, the prevalence of MSDs was 92%, while in South Africa, it was 13%. It is estimated that more than 7500 persons lose their lives in workplace accidents each day. The Global Burden of Disease 2017 study states that although the number of deaths in sub‐Saharan Africa is decreasing, the second most common cause of years lost due to disability is MSDs. A total of 7.5% of occupational disorders are related to the musculoskeletal systems, according to findings by the Social Security Institution. As stated by WHO, the most prevalent health problem (69.64%) was MSD [7]. In 2015, lower back and neck pain were the most common cause of MSD worldwide in almost all countries [8]. It is figured out that occupational risk factors account for 37% of LBP cases worldwide [9]. In global aspects among all exporters of clothes, Bangladesh is one of the biggest groups, where around 4500 garment factories and nearly 4 million people are working regularly [3, 10]. Most of the garments are located in Savar and Gazipur regions, and the methods of working in factories are mostly standing and sometimes sitting. A study conducted in Savar region found that workers are involved in both standing and sitting methods; among them, 23.4% were suffering from low back discomfort, as well as another study that noticed larger portion of workers have experienced both low back pain and neck pain [11]. According to [3, 12], long‐term job experience is associated with a higher prevalence of MSD (*p* < 0.05) and awkward posture.

MSD is a major public health issue in both developed and emerging nations. It can cause people to be unable to work, absenteeism, or even have to switch jobs, all of which have big financial costs and negative effects on their quality of life [13]. Assessing and preventing MSD risks are essential in public health because they promote workers’ health and prevent accidents at work. Reduces absences and ongoing discomfort, which improves productivity. A public health expert can also teach workers about health, which is important for better the health of the community and lowering economic loss. MSD and Rapid Entire Body Assessment (REBA) investigations give vital information on ergonomic risk factors, which support public health surveillance, policy development, and workplace reforms to reduce occupational health issues. Public health programs that integrate REBA assessments can identify high‐risk groups, ensuring that preventative actions reach the most vulnerable people.

Bangladesh's garment industry is the backbone of economic growth, providing employment opportunities to millions of people and contributing to the country's export earnings [4]. Most of them work here in a sitting position or standing position. In Bangladesh MSD is very negligible topic. Barely any work has been done in this field, in Bangladesh. Most of the workers are unaware of their health. Maximum involvement in self‐medication from the nearest pharmacy and quack. After that, they are suffering from long‐term NCDs and MSD. This study was conducted to increase the productivity and awareness among RMG workers. This study aims to measure postural risk among RMG workers through Nordic Musculoskeletal Questionnaire (NMQ) and REBA scales as well as to explore connected/responsible risk factors that is associated with MSD (in upper and lower body perspectives). This study is unique as it combines self‐reported musculoskeletal outcomes (NMQ) and objective ergonomic risk assessment (REBA) in the same group of garment workers in Bangladesh, in spite of the fact that prior studies have also evaluated WMSDs in garment workers in Bangladesh. Moreover, the present research uses the multivariable regression modeling to measure independent risk factors. The results have offered policy‐relevant information on gender‐sensitive ergonomic intervention and occupational health intervention reform in Bangladesh's largest export sector.

## 2 Methodology

### 2.1 Study Design

A cross‐sectional study has been done from July 2025 to December 2025 for this research. Both male and female garment workers’ data were collected. For this study, we have collected data from garment workers aged from 18 years to above who had at least 1 year of work experience. Workers who were physically disabled and those with chronic disease were not included in this study. Pregnant female workers and those who were absent from work for long time during data collection of time were also not included in this study.

### 2.2 Study Area

**FIGURE 1:**
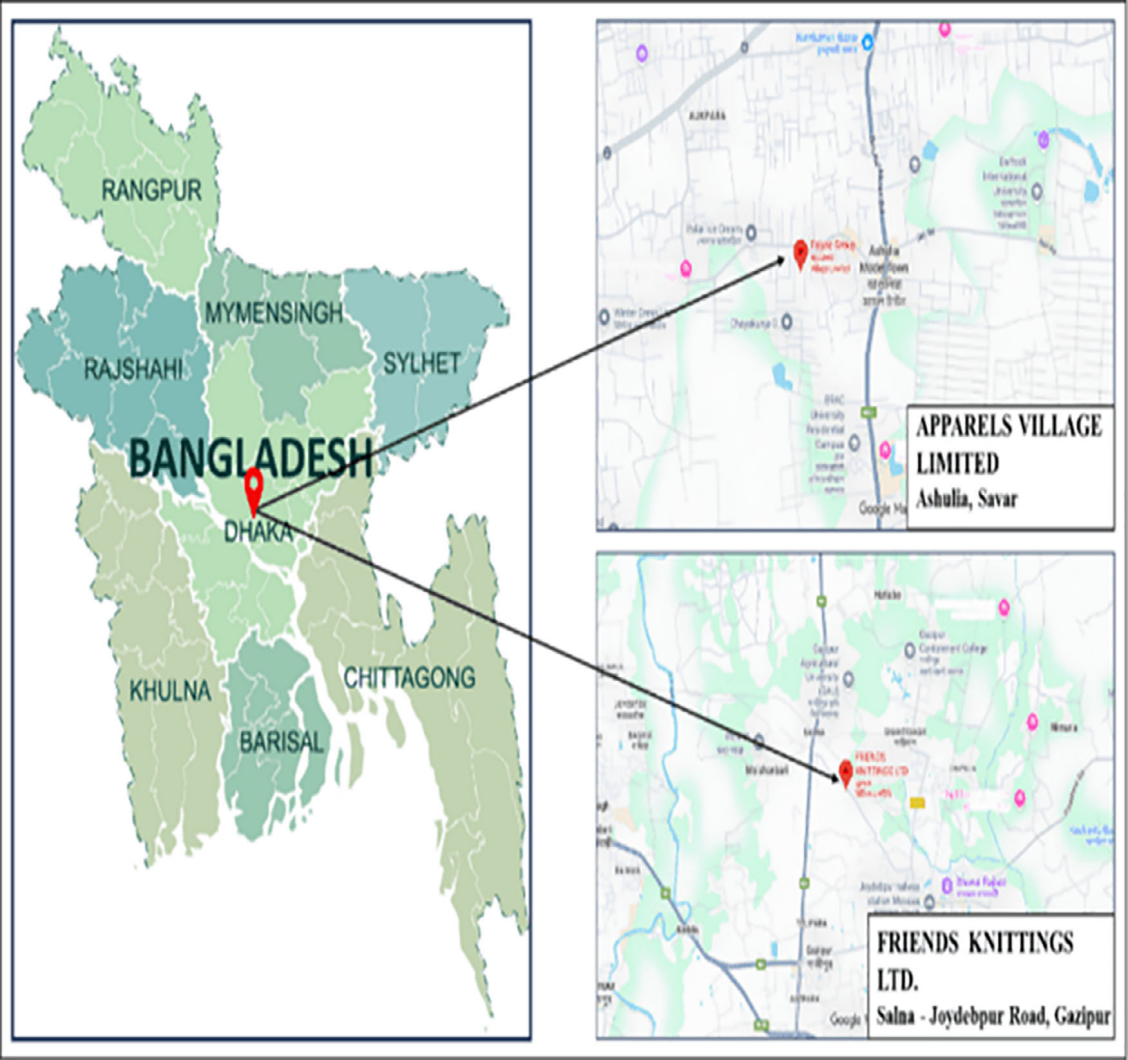
Location of data collection area.

For this study, two important areas, such as Savar and Gazipur (in Figure 1), were selected. As it is known to all, Savar and Gazipur are two of the most abundant garment's areas in Bangladesh [14]. “Apparels Village Limited” (23°52′29.47902″ N, 90°18′20.28828″ E) was selected from Savar, and “Friends Knitting Limited” (24°1′10.29052″ N, 90°23′42.16444″ E) was selected from Gazipur.

#### 2.2.1 Sampling Procedure and Sample Size

Multiphase sampling method was used. To start with, two garment factories were deliberately chosen within large industrial regions (Savar and Gazipur) because of a large concentration of workers and their availability. The selected factories were Apparels Village Limited (Savar) and Friends Knitting Limited (Gazipur). The number of workers in the chosen factories was about 2450 workers (1350 in Savar and 1100 in Gazipur). As participants were recruited from two factories, potential clustering effects were considered. However, as the number of clusters was small (*n* = 2), multilevel modeling was not feasible. Therefore, standard logistic regression was performed, and findings should be interpreted with caution regarding between‐factory variability.

The samples were proportionately allocated in the second stage to decide how many people of each factory would participate. Lastly, individuals were selected by using simple random sampling through new worker lists that the factory management provided.

The formula used in calculating the required sample size is Cochran:

$$n=\frac{Z^{2}pq}{d^{2}}.$$

where *Z* = 1.96 (95% confidence level), *p* = 0.50 (assumed prevalence because of variances in past research), *q* = 1 − *p*, and *d* = 0.04 (margin of error).

The minimum sample size calculated was 600. The final sample size required was 660 after having toised a 10% nonresponse rate. The final analysis (response rate 99.4) involved 656 complete responses.

### 2.3 Materials

#### 2.3.1 REBA Subsample Selection

**FIGURE 2:**
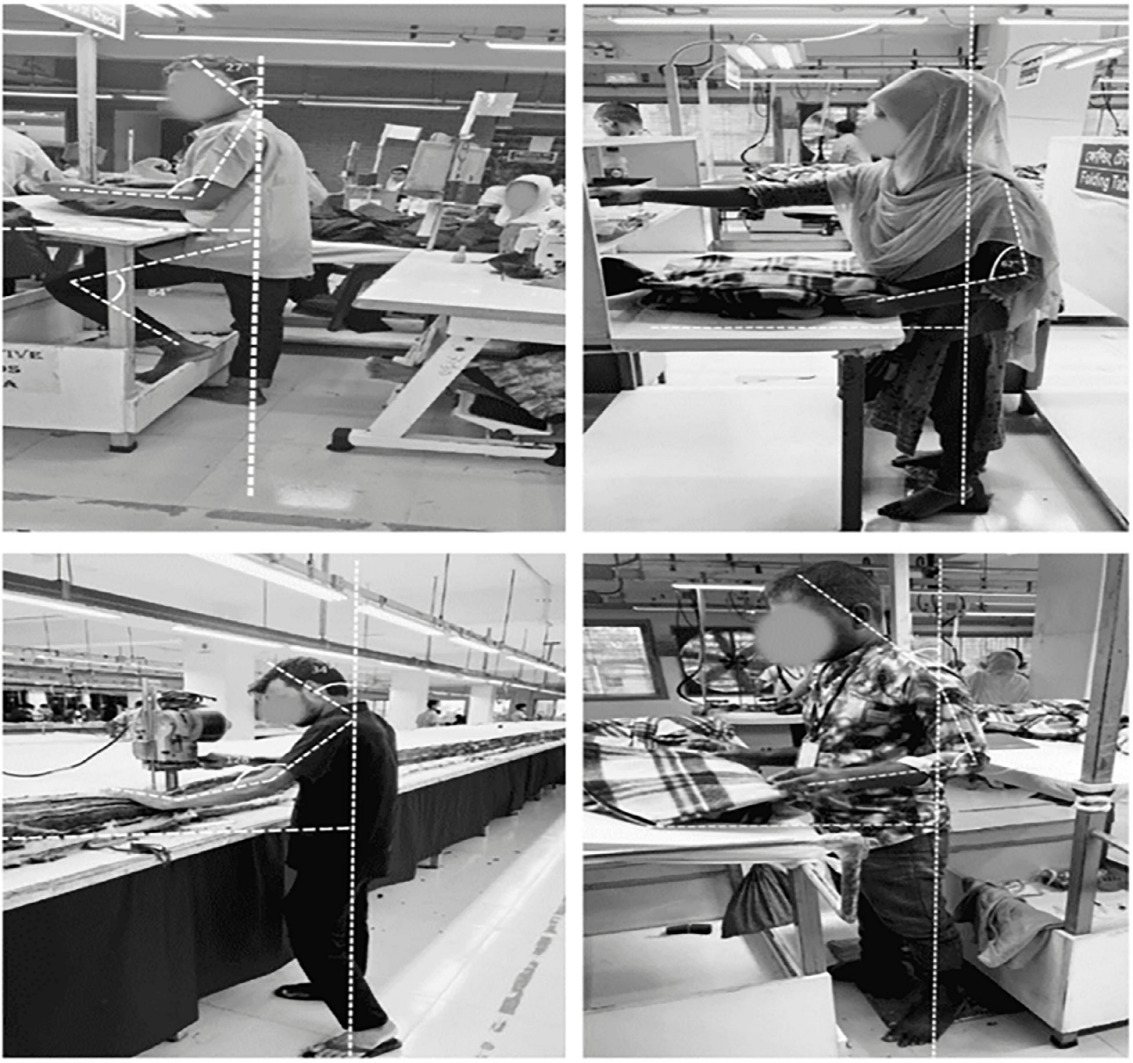
Cutting section operator adapting an awkward posture—angles marked for analysis.

**FIGURE 3:**
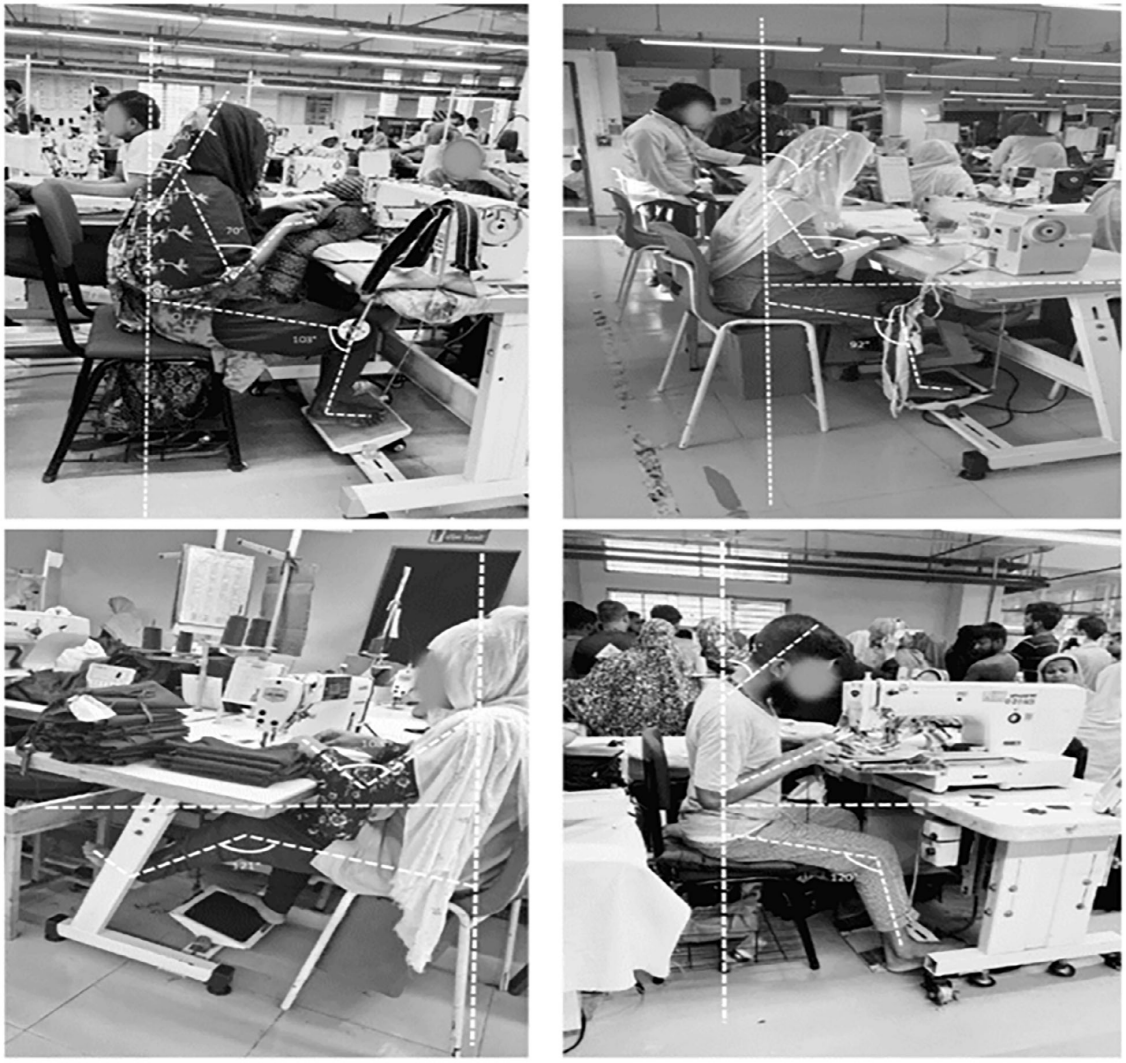
Sewing section operator adapting an awkward posture—angles marked for analysis.

REBA assessment requires detailed observational analysis and, therefore, could not be practically applied to the entire study population. For this reason, a subsample of 43 workers was selected for ergonomic posture evaluation. Participants for the REBA assessment were selected from sewing and cutting sections because these departments involve the highest exposure to repetitive tasks and prolonged static postures. Stratified sampling based on work section was used to ensure that the subsample represented the major job tasks performed in the factories. Each selected worker was observed during routine work (Figures 2 and 3) activities for approximately 10–15 min, and the most frequently adopted working posture during task performance was recorded and scored following the standard REBA protocol. The REBA findings are, therefore, presented as an exploratory ergonomic risk assessment for high‐exposure tasks rather than a representative estimate for the entire worker population.

### 2.4 Statistical Analysis

Data were entered into Microsoft Excel and analyzed using IBM SPSS Statistics version 25. Descriptive statistics were used to summarize participant characteristics and prevalence of musculoskeletal symptoms. Categorical variables were presented as frequencies and percentages. Associations between musculoskeletal symptoms and demographic or occupational variables were examined using the chi‐square test. Variables that were conceptually relevant based on previous literature and those showing associations in bivariate analysis were included in multivariable binary logistic regression models. Separate regression models were developed for neck, lower back, knee, and ankle symptoms reported during the past 12 months. Adjusted odds ratios (AORs) with 95% confidence intervals (CIs) were reported. Multicollinearity among predictors was assessed using variance inflation factor (VIF), and model fit was evaluated using the Hosmer–Lemeshow goodness‐of‐fit test. Statistical significance was considered at *p* < 0.05. Because participants were recruited from two factories, potential clustering effects at the factory level were considered. However, due to the small number of clusters (*n* = 2), multilevel modeling was not feasible. Therefore, regression results should be interpreted cautiously as potential unmeasured between‐factory variability may exist.

### 2.5 Ethical Clearance

The Institutional Review Board of the Department of Public Health was also approached to provide its ethical approval to the study (Approval No: DIU‐PH‐2025‐07). All participants were given an informed consent written.

A demographic profile (Table 1) of the participants shown in Table 2 indicates that most of them were female (54.3%), and in terms of age, most respondents’ ages are 18–25 (47.4%). In this study, the marital status of the respondents shows that most were married (87%). A majority of participants (55.6%) reported medium‐sized families consisting of three to four people. A significant portion had finished secondary school (45.4%). A large portion of participants had normal BMI (68.1%), and 16.5% had overweight. Participants work here from 1 to 10+ years. Among them, more than half (56.7%) had 1–3 years of work experience, and the majority (82.8%) worked primarily in the sewing department and rest of the cutting department. A total of 88.1% reported regularly working overtime, and the majority (67.5%) worked 9–10 h a day, which is considered overtime, and the extra overtime percentage is 88.1.

**TABLE 1:** A demographic profile of the participants (*N* = 656).

Variables	Frequency (*N* = 656)	Percentage
**Gender**		
Male	300	45.7
Female	356	54.3
**Age (year)**		
18–25	311	47.4
26–30	168	25.6
31–35	105	16.0
35+	72	11.0
**Marital status**		
Married	571	87.0
Unmarried	85	13.0
**Family members**		
1–2 persons	96	14.6
3–4 persons	365	55.6
4+ persons	195	29.7
**BMI category**		
Underweight	101	15.4
Normal	447	68.1
Overweight	108	16.5
**Educational status**		
Primary	172	26.2
Secondary	298	45.4
Higher secondary and above	186	28.4
**Work experience (year)**		
1–3	372	56.7
4–6	145	22.1
7–10	82	12.5
10+	57	8.7
**Work section**		
Sewing	543	82.8
Cutting	113	17.2
**Working hours (per day)**		
8	33	5.0
9–10	443	67.5
10+	180	27.4
**Overtime (per day)**		
No	78	11.9
Yes	578	88.1

**TABLE 2:** Rapid Entire Body Assessment (REBA) analysis procedure.

**A**	**Neck**	0°–20° = +1 >20°+ = +2 Adjust, If neck is twisted and If neck is side bending =+1			
	**Trunk**	0° = +1 0°–20° = +2 20°–60° = +3 >60°+ = +4 Adjust, if trunk is twisted and If trunk is side bending =+1	Set score in Table A and add work force		
	**Leg**	Stable = +1 Unstable = +2 Adjust, 30°–60° = +1, >60°+ = +2			Scoring 1 = negligible risk 2–3 = low risk 4–7 = medium risk 8–10 = high risk 11+ = very high risk
**B**	**Upper arm**	20° = +1 20°–45° = +2 45°–90° = +3 >90°+ = +4 Adjust, if shoulder is raised and If upper arm is abducted =+1 If arm is supported or person is leaning =−1	Set score in Table B and add coupling score	Set score from Tables A and B into Table C with activity score	
	**Lower arm**	60°–100° = +1 0°–60° = 2 >100°+ = +2			
	**Wrist**	15° = +1 >15°+ = +2 Adjust, if wrist is bent from midline or twisted =+1			

### 2.6 REBA Analysis

**FIGURE 4:**
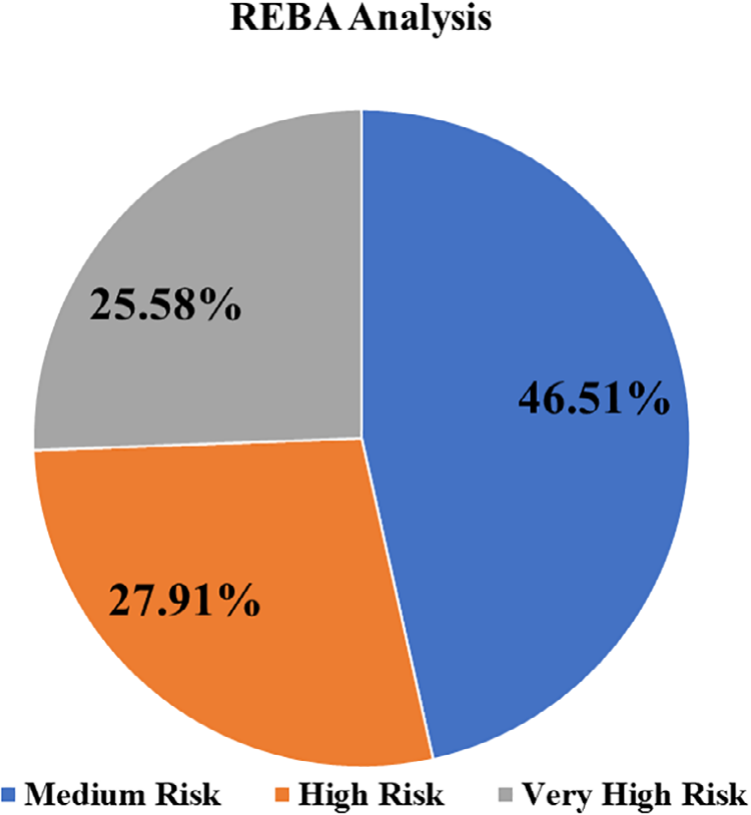
MSD risk assessment by REBA. REBA, Rapid Entire Body Assessment.

**FIGURE 5:**
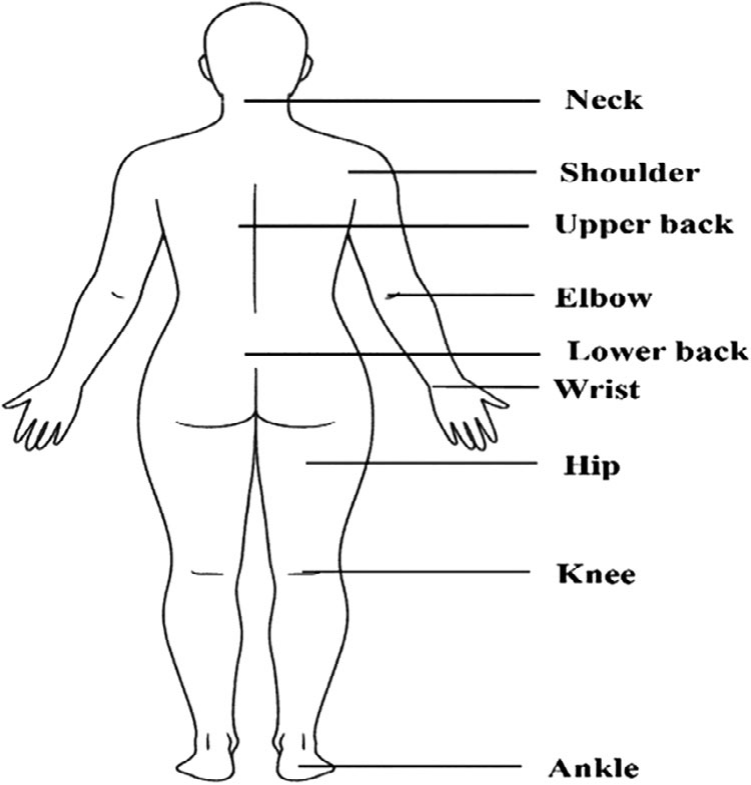
Indicating nine different body postures.

The REBA assessment was conducted by two trained public health researchers who received prior training on ergonomic posture evaluation using the standard REBA guideline. Observers independently evaluated workers’ postures during routine tasks and discussed scoring discrepancies to reach a consensus. This standardized approach helped reduce observer bias during posture evaluation. During observation, the posture most frequently maintained during the worker's primary task was recorded as the representative posture for REBA scoring. When multiple postures were observed, the posture associated with the greatest ergonomic load was selected to ensure conservative risk estimation. Figures 4 and 5 show the outcomes of the REBA test that 43 people took to find out how at‐risk they were for physical problems at work. Out of all people who participated, 20 (46.51%) were found to be at a medium risk level (REBA score 4–7). This means that some changes need to be made their posture to lower their risk of musculoskeletal pain. Moreover, 27.91% of the participants were at High Risk (REBA score 8–9), which means that ergonomic changes need to be made right away. A total of 25.58% were found to be at a very high risk (REBA score 11 or higher), which means that immediate action is needed to stop joint disorder. Overall, the results show that a lot of the participants are exposed to moderate to very high ergonomic risks. This shows how important it is to immediately assess and make ergonomic changes in the workplace to protect health and safety at work.

Table 3 presents the association between demographic and occupational variables and the occurrence of neck and lower back symptoms among the participants. In this analysis, musculoskeletal symptoms (neck and lower back problems) were considered the outcome variables, whereas demographic and occupational characteristics, such as gender, marital status, work section, posture, working hours, overtime, and work experience, were treated as predictor variables. Neck pain/cervical strain is mainly influenced by working hours and overtime as the findings indicate that 74.7% and 19.9% of workers who work more than working hours (8 h) were facing neck pain, and it is statistically significant as *p* value 0.009 < 0.05. Among those whose workers were involved in overtime, 91.9% of the workers were facing cervical strain, and it is also statistically significant (as *p* value 0.035 < 0.05). Long‐term overtime can produce severe cervical strain to the workers, which intact severe nerves (cervical spinal nerves, C1–C8) and muscles (trapezius, levator scapulae, and sternocleidomastoid) injuries in the long term. Moreover, neck pain is also severe in female (58.4%) and married (85.5%) workers in sewing section (80.5%) and full‐time setting position (77.4%) and working experience (54.3%). But this variable is not statistically significant as *p* value >0.05. In terms of lower back, it is very common, and all the variables are statistically significant with this problem. Workers who work overtime have higher rates of lower back pain (92.0%) than those who work regular hours (8 h), indicating that overtime is the primary cause of this condition, and it is statistically noticeable (*p* value 0.010 < 0.05). Similarly, the prevalence was higher among married participants (90.5%) than unmarried ones (*p* value 0.025 < 0.05). A large portion of females (65.0%) reported lower back problems compared to males, and it is statistically significant (*p* < 0.001). Workers in the sewing section (89.1%) exhibited a higher incidence of lower back pain (*p* < 0.001). Regarding posture, respondent who work full time sitting position (82.8%) were more affected than those who work standing and in both position, and it is significantly noticeable (*p* < 0.001). Furthermore the prevalence of lower back pain increased with work experience and working hours, 49.6% being highest among those with 1–3 years of experience (*p* value 0.007 < 0.05) and (63.5%) those who work 9–10 h/day, and it is statistically significant with *p* < 0.001.

**TABLE 3:** Association between neck and lower back problem with demographic and occupational variable.

Variables		Facing neck problems			Facing lower back problems		*χ* ^2^ (*p* value)
		Yes *n* (%)	No *n* (%)	*χ* ^2^ (*p* value)	Yes *n* (%)	No *n* (%)	
Gender	**Male**	92 (41.6)	208 (47.8)	2.260 (0.133)	125 (35.0)	175 (53.4)	**21.687 (0.000)** ^***^
	**Female**	129 (58.4)	227 (52.2)		148 (65.0)	208 (46.6)	
Marital status	**Married**	189 (85.5)	382 (87.8)	0.685 (0.408)	248 (90.5)	323 (84.6)	**5.018 (0.025)** ^***^
	**Unmarried**	32 (14.5)	53 (12.2)		26 (9.5)	59 (15.4)	
Section	**Sewing**	178 (80.5)	365 (83.9)	1.164 (0.281)	244 (89.1)	299 (78.3)	**13.001 (<0.001)** ^***^
	**Cutting**	43 (19.5)	70 (16.1)		30 (10.9)	83 (21.7)	
Sitting position	**Sitting**	171 (77.4)	317 (72.9)	1.745 (0.418)	227 (82.8)	261 (68.3)	**21.511 (<0.001)** ^***^
	**Standing**	33 (14.9)	82 (18.9)		38 (13.9)	77 (20.2)	
	**Both**	71 (7.7)	36 (8.3)		9 (3.3)	44 (11.5)	
Working experience	**1–3 years**	120 (54.3)	252 (57.9)	2.771 (0.428)	136 (49.6)	236 (61.8)	**12.270 (0.007)** ^***^
	**4–6 years**	57 (25.8)	88 (20.2)		73 (26.6)	72 (18.8)	
	**7–10 years**	25 (11.3)	57 (13.1)		34 (12.4)	48 (12.6)	
	**10+ years**	19 (8.6)	38 (8.7)		31 (11.3)	26 (6.8)	
Working hour	**8 h**	12 (5.4)	21 (4.8)	**9.501 (0.009)** ^***^	7 (2.6)	26 (6.8)	**14.114 (<0.001)** ^***^
	**9–10 h**	165 (74.7)	278 (63.9)		174 (63.5)	269 (70.4)	
	**10+ h**	44 (19.9)	136 (31.3)		93 (33.9)	87 (22.8)	
Overtime	**Yes**	203 (91.9)	18 (8.2)	**4.463 (0.035)** ^***^	252 (92)	22 (8.0)	**6.696 (0.010)** ^***^
	**No**	375 (86.2)	60 (13.8)		326 (85.3)	56 (14.7)	

^***^ This means statistical Significance, *p* value < 0.05, (95% CI).

Table 4 shows the association between demographic and occupational variables and lower body musculoskeletal symptoms (knee and ankle problems). In this analysis, knee and ankle symptoms were considered outcome variables, whereas demographic and occupational characteristics were treated as explanatory variables. Prolonged static posture and repetitive work activities may contribute to musculoskeletal discomfort among garment workers.

**TABLE 4:** Association between knee and ankle problem with demographic and occupational variable.

Variables		Facing knee problems			Facing ankle problems		*χ* ^2^ (*p* value)
		Yes *n* (%)	No *n* (%)	*χ* ^2^ (*p* value)	Yes *n* (%)	No *n* (%)	
Gender	**Male**	52 (38.4)	248 (47.2)	2.931 (0.087)	10 (43.0)	192 (47.3)	1.118 (0.290)
	**Female**	60 (61.6)	296 (52.8)		127 (57.0)	229 (52.7)	
Marital status	**Married**	100 (89.3)	471 (86.6)	0.602 (0.438)	198 (84.3)	373 (88.6)	2.522 (0.112)
	**Unmarried**	12 (10.7)	73 (13.4)		37 (15.7)	48 (11.4)	
Section	**Sewing**	88 (78.6)	455 (83.6)	1.673 (0.196)	173 (73.6)	370 (87.9)	**21.536 (<0.001)** ^***^
	**Cutting**	24 (21.4)	89 (16.4)		62 (26.4)	51 (12.1)	
Sitting position	**Sitting**	80 (71.4)	408 (75.0)	**7.855 (0.020)** ^***^	142 (60.4)	346 (82.2)	**58.816 (<0.001)** ^***^
	**Standing**	28 (25.0)	87 (16.0)		77 (32.8)	38 (9.0)	
	**Both**	4 (3.6)	49 (9.0)		16 (6.8)	37 (8.8)	
Working experience	**1–3 years**	55 (49.1)	317 (58.3)	4.751 (0.191)	128 (54.5)	244 (58.0)	4.350 (0.226)
	**4–6 years**	26 (23.2)	119 (21.9)		62 (26.4)	83 (19.7)	
	**7–10 years**	20 (17.9)	62 (11.4)		28 (11.9)	54 (12.8)	
	**10+ years**	11 (9.8)	46 (8.5)		17 (7.2)	40 (9.5)	
Working hour	**8 h**	7 (6.3)	26 (4.8)	1.161 (0.560)	8 (3.4)	25 (5.9)	**7.410 (0.025)** ^***^
	**9–10 h**	71 (63.4)	372 (68.4)		174 (74.0)	269 (63.9)	
	**10+ h**	34 (30.4)	146 (26.8)		53 (22.6)	127 (30.2)	
Overtime	**Yes**	104 (92.9)	8 (7.1)	2.905 (0.088)	215 (91.5)	20 (8.5)	**3.992 (0.046)** ^***^
	**No**	474 (87.1)	70 (12.9)		363 (86.2)	58 (13.8)	

^***^ This means statistical Significance, *p* value < 0.05, (95% CI).

Binary logistic regression was used to examine factors that are independently related to the occurrence of MSDs in four body parts (neck, lower back, knee, and ankle), which are shown in Table 5. The odds ratios (ORs) that reported were adjusted to gender, marital status, work section, posture, work experience, working hours, and overtime. VIF was also used to measure multicollinearity, and no significant multicollinearity was identified (VIF < 2.5). The Hosmer–Lemeshow test was used to evaluate model goodness of fit (*p* > 0.05) and showed that the model fits well. Females were at significantly higher risk of developing lower back (OR: 2.017, 95% CI: 1.395–2.917), knee (OR: 1.78, 95% CI: 1.097–2.889), and ankle (OR: 2.025, 95% CI: 1.346–3.046) disorders compared to males. No significant association was observed for neck disorders. No statistically significant associations were found between marital status (married vs. unmarried) and MSDs in any region. Employees in the sewing section had a significantly lower risk of neck disorders (OR: 0.460, 95% CI: 0.222–0.955) compared to those in the cutting section. No significant associations were observed for other regions. Working with fully setting posture was significantly associated with increased odds of lower back (OR: 3.04, 95% CI: 1.284–7.195), knee (OR: 4.475, 95% CI: 1.42–14.101), and ankle (OR: 5.783, 95% CI: 2.731–12.247) problems, compared to those who worked in both postures. On the other hand, standing posture was significantly associated with lower odds of ankle disorders (OR: 0.608, 95% CI: 0.298–1.241). Workers who have 4–6 years of experience were significantly associated with an increased risk of lower back (OR: 1.982, 95% CI: 1.206–2.746) and ankle disorders (OR: 1.737, 95% CI: 1.132–2.665). Experience of 7–10 years was significantly associated with increased knee disorders (OR: 2.18, 95% CI: 1.191–3.989). A total of 10+ years was significantly associated with lower back disorders (OR: 2.44, 95% CI: 1.322–4.507). The likelihood of neck and knee disorders was lower among workers who worked 9, 10, or 10 h or greater hours as opposed to 8 h (OR, 0). This can be an indicator of a healthy worker effect in that worker who can work longer hours are less symptomatic or better adapted. Nevertheless, there were independent but non‐significant associations between overtime work and increased odds of knee, neck, and ankle disorders, implying that cumulative overtime work and not hours of work may also raise the risk of MSD. Moreover, significant risk factors for MSDs in various anatomical regions included work sections, gender, years of working experiences, working posture, overtime, and working hour per day. Finally, these findings suggest that ergonomic interventions and job design modifications, especially for female workers and those exposed to prolonged sitting, overtime, and specific work sections, may help reduce the burden of MSDs in this population.

**TABLE 5:** Binary logistic regression among different variable.

		OR (95% CI)			
Factors		Neck	Lower back	Knee	Ankle
Gender	Female/Male	1.30 (0.89–1.90)	2.01 (1.40–2.92)^*^	1.78 (1.10–2.89)^*^	2.02 (1.35–3.05)^*^
Marital status	Married/Unmarried	0.75 (0.45–1.27)	1.14 (0.67–1.97)	1.21 (0.60–2.46)	0.86 (0.50–1.50)
Section	Sewing/Cutting	0.46 (0.22–0.96)^*^	1.43 (0.70–2.91)	0.86 (0.36–2.06)	1.46 (0.73–2.95)
Working posture *Both (ref.)*	Sitting	0.68 (0.32–1.46)	3.04 (1.28–7.20)^*^	4.47 (1.42–14.10)^*^	5.78 (2.73–12.25)^*^
	Standing	1.69 (0.80–3.59)	2.60 (1.20–5.99)^*^	2.06 (0.67–6.39)	0.60 (0.30–1.24)
Working experience	4–6	1.40 (0.93–2.12)	1.98 (1.21–2.75)^*^	1.40 (0.83–2.38)	1.73 (1.13–2.67)^*^
	7–10	1.01 (0.59–1.72)	1.25 (0.76–2.09)	2.18 (1.19–3.99)^*^	1.20 (0.70–2.07)
	10+	1.37 (0.73–2.59)	2.40 (1.32–4.51)^*^	1.77 (0.83–3.81)	1.32 (0.68–2.60)
	***1*–*3 (ref.)***	—			
Working hour	9–10	0.29 (0.10–0.86)^*^	1.49 (0.52–4.27)	0.12 (0.02–0.64)^*^	1.13 (0.38–3.36)
	10+	0.14 (0.05–0.47)^*^	2.24 (0.74–6.86)	0.14 (0.03–0.82)^*^	0.80 (0.25–2.59)
	***8 (ref.)***	—			
Overtime	No/Yes	3.62 (2.55–8.45)^*^	1.70 (0.88–3.31)	6.40 (1.42–28.89)^*^	2.34 (1.11–4.99)^*^

* *p* value <0.05, (95% CI).

According to the NMQ results in Table 6, which cover nine body parts—neck, shoulder, upper back, elbow, wrist, lower back, hip, knee, and ankle—data were collected on problems experienced during the past 7 days, problems affecting daily activities over the past 12 months; and doctor visits within the past 12 months. In the previous 12 months, a considerable proportion of participants experienced pain or discomfort in the lower back (39.5%), neck (33.5%), and ankle (33.5%). The knee was also moderately affected (15.9%). However, only a small percentage of respondents reported issues in the elbow (2.1), wrist (2.9), and upper back (2.9), indicating that these areas were comparatively less prone to musculoskeletal problems. In contrast, within the past 7 days, the most frequently affected body regions are the lower back (35.7%), ankle (31.1%), and neck (19.1%). Conversely, the least affected regions included the elbow (1.7%), upper back (2.9%), and wrist (2.9%). During the past 12 months, difficulties in performing daily activities are most frequently reported for the neck (11.6%), ankle (11.6%), and lower back (11.4%). Over the last 12 months, lower back pain led to more doctor visits (15.7%) compared to ankle (12.8%) and neck pain (12.3%).

**TABLE 6:** Result of musculoskeletal disorders from Nordic Musculoskeletal Questionnaire (NMQ).

MSD	Facing problems past 7 days *n* (%)	Facing problems past 12 months *n* (%)	Facing problems past 12 months daily activities *n* (%)	Doctor visit past 12 months *n* (%)
Neck	125 (19.1)	220 (33.5)	76 (11.6)	81 (12.3)
Shoulder	55 (8.4)	63 (9.6)	19 (2.9)	26 (4.0)
Upper back	19 (2.9)	31 (4.7)	9 (1.4)	11 (1.7)
Elbow	11 (1.7)	14 (2.1)	1 (0.2)	8 (1.2)
Wrist	19 (2.9)	18 (2.7)	6 (0.9)	9 (1.4)
Lower back	234 (35.7)	259 (39.5)	75 (11.4)	103 (15.7)
Hip	55 (8.4)	57 (8.7)	20 (3.0)	18 (2.7)
Knee	91 (13.9)	104 (15.9)	24 (3.7)	33 (5.0)
Ankle	204 (31.1)	220 (33.5)	76 (11.6)	84 (12.8)

Abbreviation: MSD, musculoskeletal disorder.

## 3 Discussion

Because this study used a cross‐sectional design, causal relationships between occupational exposures and musculoskeletal symptoms cannot be established. The observed associations should, therefore, be interpreted as correlations rather than causal effects. This study provides the prevalence and risk factors of WMSDs in comprehensive way among RMG workers in the Savar and Gazipur areas of Bangladesh. Applying both the NMQ and the REBA scales, our study found a high burden of WMSDs, especially in the lower back, neck, and ankle, echoing previous study in similar occupational settings [1, 9]. In this study, most of the participants, around 39.5%, suffered from lower back pain in the last 12 months with neck 33.5% and ankle 33.5% too. These findings are consistent with previous studies in Bangladesh and globally, which have identified the lower back as the most commonly affected region among both of industrial and garments workers [9, 10, 13]. Physical demands and ergonomic challenges inherent in garments factory work lead to the high prevalence of MSDs in the lower extremities and back. The risk for lower back, knee, and ankle problems is significantly higher in females compared to males (ORs: 2.017, 1.78, and 2.025, respectively); similar findings were found that women in industrial settings are more susceptible to certain WMSDs [9, 15]. Prevalence of lower back and ankle MSDs is mainly observed among workers who worked in sewing sections because of repetitive task and prolonged sittings. Same findings were observed in another study and they found that both highlighted the ergonomic hazards associated with sewing activities [2, 16]. The full body posture analysis by REBA tools and the findings was around 46.5% respondent at in medium risk, 27.9% at high risk and rest 25.6% at very high risk. This result focuses on the emergency need for ergonomic interventions as similar findings were found a significant portion of RMG workers at moderate to high risk for posture of MSDs injury [2, 6]. Neck pain and back pain were strongly associated with both of prolonged sitting and overtime regularly. Workers who reported being involved in regular overtime had significantly higher odds of experiencing these conditions (OR for neck pain 3.62, for knee pain 6.40, and for ankle problems 2.35). This study findings are strongly associated with previous literature, which points out long working hours, fixed or static posture and repetitive motions as major contributor to most of MSDs [9, 10]. Workers who were involved with 4–6 years of work experience had higher risk for lower back pain and ankle pain, whereas those who were involved with 7–10 years are more likely to experience knee pain. These findings align with another study about understanding that cumulative exposure to occupational hazards increases the risk of MSDs [3, 7]. Surprisingly, those who worked longer hours (more than 8 hours per day) had lower odds of knee and neck pain. This may be because they take intermittent breaks to rest before continuing work. Additionally, this finding may reflect the healthy worker effect, where individuals who are able to sustain longer working hours tend to be less symptomatic or have adapted to the work demands [17]. However, some variables were strongly associated with overtime and work‐related MSDs. To solve these issues immediate workplace interventions like regular break, ergonomic redesign and worker education and training on safe work practice. The authority of public health and industry management should give priority the implementation of some ergonomic assessment such as REBA, as these tolls are helpful to identify high risk group and inform higher authority for intervention [1]. Furthermore, the findings highlight the importance of gender‐sensitive interventions, as female workers are disproportionately affected. Addressing these issues is crucial not only for improving worker health and well‐being but also for maintaining productivity in a sector critical to Bangladesh's economy [13, 17].

### 3.1 Limitations

This study is limited by its design—cross‐sectional—which restricts causal inference. Moreover, recall bias can be an issue due to self‐reported data. Despite these limitations, the study adds robust evidence to the literature on occupational health risks in Bangladesh's RMG sector. Another limitation of the present study is that data were collected from only two garment factories located in Savar and Gazipur using purposive factory selection. Although these locations represent major industrial areas of Bangladesh's RMG sector, the findings may not fully represent all RMG factories across the country. Therefore, the results should be interpreted with caution regarding generalizability to other industrial settings. The study achieved a high response rate (99.4%), which may partly reflect the structured data collection process conducted during working hours with the cooperation of factory management. Although this helped minimize nonresponse bias, it is possible that some workers may have felt encouraged to participate due to workplace dynamics, which could introduce potential participation bias. The study relied on self‐reported musculoskeletal symptoms using the NMQ, which may be subject to recall bias. In addition, the possibility of a healthy worker effect cannot be excluded, as workers with severe symptoms may have already left employment. Although the REBA assessment was performed on a smaller subsample, the ergonomic observations still provide useful contextual information regarding postural risks in high‐exposure tasks within garment production. However, these findings should be interpreted as exploratory rather than population‐representative estimates.

## 4 Conclusion

This research indicates that the WMSDs are high among the RMG workers in Bangladesh, especially affecting lower back, neck, and ankle. Women workers, prolonged sitting position, exposure to overtime and cumulative work experience were all important predictors of WMSDs. The results of the REBA also demonstrated that a significant percentage of workers were exposed to the moderate‐to‐very high levels of ergonomic risks, which is why there is an urgent necessity for the ergonomic redesign of the workplace. Structured ergonomic interventions, regulated use of overtime, and occupational health gender‐sensitive interventions are essential in preventing the burden of WMSD and maintaining the productivity of the workforce in the garment industry in Bangladesh. It is suggested that longitudinal research should be conducted in the future to determine causal associations and assess the effectiveness of interventions.

## Author Contributions

**Md. Mehedi Hasan**: conceptualization; methodology; data curation; writing – original draft. **Jannatul Hayat Rima**: conceptualization; methodology; formal analysis; data curation; writing – original draft. **Hafsa Hasan Jannat**: conceptualization; methodology; software; formal analysis; data curation. **Md. Yeasir Arafath Jisan**: software; formal analysis; data curation; writing – original draft. **Md. Nazmul Haque Nayem**: data curation; writing – original draft. **Md. Mejbah Uddin Mithu**: conceptualization; validation; supervision; visualization; project administration; resources; writing – review and editing.

## Funding

The authors have nothing to report.

## Conflicts of Interest

The authors declare no conflicts of interest.

## Data Availability

The datasets generated and analyzed during the current study are available from the corresponding author upon reasonable request.
